# Account of Some Curiosities in Natural History

**Published:** 1817-07

**Authors:** John Hoare

**Affiliations:** Surgeon and Apothecary.


*For the London Medical and Physical Journal.*

*Account of some Curiosities in Natural History;* by JOHN HOARE, Esq. Surgeon and Apothecary.

IN the month of August 1816, as some men were quarrying stones, in the garden of Mr. John Daniell, land-surveyor of Warminster, they discovered, in the middle of a vast stratum of rock, and at nearly twelve feet from the surface, a toad and a newt. Both animals were alive, and of full size; their habitation was just large enough to contain them—the interior was perfectly smooth, lined with sand, and without the smallest orifice or crack. On being exposed to the air, the colour of both animals altered, and life for a few moments was suspended: they revived, and lived for about four hours, exhibiting occasionally symptoms of pain and convulsive motions about the throat. Their mouths were glued together, insomuch that the violent strugglings which the putting them into spirits of wine occasioned did not open them, or dissolve the glutinous matter. After they were dead, with difficulty I forced open their mouths. How long they might have lived I cannot tell, but probably not long, as they continued torpid and inactive four hours after their exposure; when inconsiderately, and from their hideous appearance, I put a period to their existence by immersion in alkohol; but they struggled hard, and survived long even here. The eyes of both were perfect and sensible, especially the toad's, whose iris was variegated beautifully, and suffered contraction and dilatation on the application of light, as similar to the human eye. It was proved to be a water-newt, for, being left together in a pan in the open air, a storm came on, which made the newt quite lively. He exhibited signs of great antipathy to his long and close companion, and endeavoured to get out of the pan. This newt is larger than the common ones, and seeded on the back in the manner of the green lizard, with vermilion and black spots on the belly.

Some medical and other gentleman, as well as myself, examined the place, and all the circumstances connected herewith. It is the decided opinion of all, that they could not possibly have had the least atmospheric air, and we are certain the stratum in which they were found was in a *state of nature, unaltered ever.*

Near the same place, but in the stratum of sand *following,* (for there were alternate strata of sand and rock-stone to this depth) there were also found some shark's teeth, and also a tooth of the alligator, in a state of high preservation;

 likewise likewise bones, apparently the clavicle and scapula of a human subject, but I am informed they are bones of the crocodile. These, on being exposed to the air, almost immediately pulverised. There were also found a variety of other marine substances, in a state of fossilization.

The above curiosities I have in my possession. The toad and newt are preserved in spirits; but it is worthy of remark, that the cuticle of the toad which the air, on exposure, turned nearly black, is scaling off, leaving to view the original colour, which is also the colour of the rock-stones out of which they were dug.

The above being a recent discovery, and of the clearest evidence, I think will prove worthy to be noticed in your department of natural history; and, as you solicited, in a late Number, materials of this nature, I feel pleasure in transmitting it to you,—at the same time indulging myself with the hope that some of your intelligent readers will not fail to throw some light on this interesting branch of oryctology, and, in reference to the animals, I had almost said *immortality*.

Dr. Wilkinson, of Bath (Philosophical Society), among other comments on the subject, remarked, that this lizard was the first ever noticed or found in this condition. This led to the communication of two lizards found in a chalk-bed, and the interesting remarks thereon, by the Rev. Mr. Powell, of Weeton Parsonage, Brandon, Suffolk. Doubtless, more circumstances of this nature are in existence, but not made known, and, perhaps, many more are overlooked from inattention, or a want of interest in the subject.

*Warminster; May* 27, 1817.

